# Synthesis of Copper(II) Trimesinate Coordination Polymer and Its Use as a Sorbent for Organic Dyes and a Precursor for Nanostructured Material

**DOI:** 10.3390/polym12051024

**Published:** 2020-05-01

**Authors:** Gulzhian I. Dzhardimalieva, Rose K. Baimuratova, Evgeniya I. Knerelman, Galina I. Davydova, Sarkyt E. Kudaibergenov, Oxana V. Kharissova, Vladimir A. Zhinzhilo, Igor E. Uflyand

**Affiliations:** 1 Institute of Problems of Chemical Physics of the Russian Academy of Sciences, Chernogolovka, Moscow Region 142432, Russia; roz_baz@mail.ru (R.K.B.); kge@icp.ac.ru (E.I.K.); davydova@icp.ac.ru (G.I.D.); 2 Moscow Aviation Institute (National Research University), Moscow 125993, Russia; 3 Institute of Polymer Materials and Technology, Almaty 050019, Kazakhstan; skudai@mail.ru; 4 Laboratory of Engineering Profile, Satbayev University, Almaty 050013, Kazakhstan; 5 Universidad Autónoma de Nuevo León, 66455 San Nicolás de los Garza, Nuevo León, Mexico; okhariss@mail.ru; 6 Department of Chemistry, Southern Federal University, Rostov-on-Don 344090, Russia; i06993@yandex.ru (V.A.Z.); ieuflyand@sfedu.ru (I.E.U.)

**Keywords:** copper trimesinate, coordination polymer, metal-organic frameworks, dye adsorption, isotherm, thermolysis

## Abstract

Several important synthesis pathways for metal-organic frameworks (MOFs) were applied to determine how the synthesis methods and conditions affect the structure and adsorption capacity of the resulting samples. In the present work, three different synthesis routes were used to obtain copper trimesinate coordination polymer: Slow evaporation (A), solvothermal synthesis using a polyethylene glycol (PEG-1500) modulator (B), and green synthesis in water (C). This MOF was characterized by elemental analysis, infrared spectrometry, X-ray diffraction, scanning electron microscopy, thermogravimetry and volumetric nitrogen adsorption/desorption. The samples have permanent porosity and a microporous structure with a large surface area corresponding to the adsorption type I. The obtained MOF was tested as a sorbent to remove organic dyes methylene blue (МВ), Congo red (CR) and methyl violet (MV) as examples. Dye adsorption followed pseudo-first-order kinetics. The equilibrium data were fitted to the Langmuir and Freundlich isotherm models, and the isotherm constants were determined. Thermodynamic parameters, such as changes in the free energy of adsorption (∆*G*^0^), enthalpy (∆*H*^0^), and entropy (∆*S*^0^), were calculated. Thermolysis of copper trimesinate leads to the formation of carbon materials Cu@C with a high purity.

## 1. Introduction

Currently, continued economic growth, widespread urbanization and rapid population growth are leading to ever-increasing environmental pollution [1]. Among the most acute problems, it should be noted the depletion of water resources due to their contamination by various pollutants, including inorganic and organic chemicals. Synthetic dyes are one of the main groups of water and wastewater pollutants, since they are widely used in textile, printing, paper, leather, plastic, rubber, carpet, food, pharmaceutical, and cosmetic industries [2,3]. These dyes create one of the most acute environmental problems due to their potential adverse effects on human health [4,5]. In addition, many organic dyes contain chemicals that are toxic or carcinogenic to mammals and other living organisms [6,7]. An important problem is also the non-biodegradable nature of some dyes and their resistance to light and oxidizing agents [8,9]. Methylene blue (MV), Congo red (CR) and methyl violet (MV) are some of the most common organic dyes present in wastewater and industrial effluents. Therefore, it is very important to remove dyes before discharging wastewater into water resources, in order to reduce environmental damage.

A variety of biological, chemical and catalytic methods are used to remove dyes from wastewater [10,11,12,13]. Among them, the adsorption of dyes on solids with a high specific surface area is widely used due to its high efficiency, reliability, low cost, versatility and ease of operation [14,15,16]. The most common adsorbents are activated carbon [17,18,19], polymers [20], zeolites [21] and biomaterials [22,23,24]. Among their shortcomings, low adsorption capacity and separation difficulties should be noted. Therefore, an important task is the development of effective and cost-effective adsorbents for the removal of organic dyes from wastewater, which have a large capacity, high adsorption rate and easy separation.

In this regard, the attention of researchers was attracted to the coordination polymers or metal-organic frameworks (MOFs), which consist of metal-oxo clusters or metal ions and organic linkers, and are characterized by a diverse crystalline architecture, large specific surface areas and good porosities [25,26]. Their physico-chemical characteristics can be easily controlled by adjusting the structure and functionality, as well as using post-synthetic modification to improve the adsorption properties. Studies have shown the effectiveness of using MOF to remove pollutants, in particular, organic dyes, from wastewater [27,28,29,30]. A typical example is the adsorption of organic dyes on titanium-based MOFs, MIL-125, which improves significantly after NH_2_-functionalization [31]. In another interesting study, nickel-based MOF was used to remove MB as a cationic dye [32]. It should also be noted that this study looks at the influence of the size of MOF mesopores with hierarchical pores on the adsorption capacity for various organic pollutants [33]. Zirconium-based MOFs showed high structural resistance to water, which made it possible to use them for adsorption removal of anionic and cationic dyes from aqueous solutions [34]. It is of interest to use the four cobalt (II) coordination polymers [35] and siloxane-based MOF [36] for the degradation removal of CR azo dye. Two copper (I) cyanide coordination polymers were used to decompose and remove MB [37].

MOFs attract considerable attention as precursors of nanostructured materials, since they are not only starting materials, but also stabilizers of the resulting nanoparticles [38,39,40,41,42]. One of the simplest and most rational methods for the synthesis of new nanostructured materials is solid-phase thermolysis of MOFs with various structures and compositions. Thermal decomposition of MOFs under various conditions allows one to obtain a variety of nanomaterials, for example, carbon, metals, metal oxides, etc., with the desired size and morphology.

Among the various MOFs, MOF, such as Cu_3_(BTC)_2_(H_2_O)_3_⋅xH_2_O (where BTC = 1,3,5-benzenetricarboxylate) containing the binuclear Cu(II) paddlewheel structures are of particular interest for testing the adsorption capacity. This MOF was first synthesized in 1999 [43] and designated as HKUST-1 (Hong Kong University of Science and Technology) or MOF-199. It has a high specific surface area and an interconnected 3D pore system with pore sizes of 9 Å × 9 Å [44,45]. Various Cu–BTC complexes were studied, including Cu_3_(BTC)_2__⋅_3H_2_O [46,47], Cu_2_(OH)(BTC)(H_2_O)_2_·nH_2_O [48], and Cu(BTC–H_2_)_2_(H_2_O)_2_⋅3H_2_O [49]. The synthesis of Cu-BTC is mainly based on conventional hydrothermal processes, with long reaction times and relatively high temperatures, as well as a number of processes with a continuous flow [50,51,52]. As an example, it is possible to obtain uniform hollow Cu–BTC microspheres under conditions of an interfacial reaction using a continuous droplet microreactor [50]. A high-rate of synthesis of Cu–BTC was achieved based on uniform mixing of the reactant streams using a continuous impinging jet reactor under high pressure [51]. Hollow nanocrystalline MOFs were obtained in a continuous process using a special nozzle for spraying precursor solutions to create atomized droplets [52]. High-rate synthesis of Cu–BTC MOFs with a BET surface area of more than 1600 m^2^ g^−1^ and with a yield of 97% was carried out in a continuous-flow microreactor-assisted solvothermal system with a total reaction time of 5 min [53,54]. It should be noted the preparation of Cu–BTC MOF by green synthesis with water [55]. Carboxylate-based MOFs are characterized by a regular structure and permanent porosity after thermal/vacuum activation, which allows them to encapsulate a variety of guest substrates into the bulk structure, which makes them promising solid materials for a number of applications, such as gas storage and separation.

Although considerable work has been done on the synthesis of HKUST-1, very few studies of HKUST-1 have indeed produced high-quality crystals with a large surface area. In particular, some studies reported very large surface areas, while others reported very small values using the solvothermal synthesis method. An attempt was made to improve the yield and surface area of HKUST-1 using the atmospheric pressure method [56]. However, the typical octahedral crystalline structure of HKUST-1 was not obtained. Recently, HKUST-1 has also received significant attention in wastewater treatment due to its excellent properties [57,58]. However, the effect of water adsorption on the structure of MOFs was shown [59]. For example, it was found that Cu_3_(BTC)_2_ is characterized by a low surface area, a change in crystallinity, and damage to the surface of the crystal. In addition, the stretching of the Cu_2_C_4_O_8_ cage occurs as a result of the absorption of water molecules near Cu sites [60]. To improve the adsorption properties of HKUST-1, nanoparticles of graphene, graphite, and carbon nanotubes were included in this MOF [61,62,63,64,65]. Although a number of works have been devoted to the adsorption of dyes on HKUST-1, only a few reports on HKUST-1 produced large surface areas and pore volumes. Therefore, additional studies on the synthesis and characterization of HKUST-1 are needed.

In this work, several important synthesis pathways for MOFs were applied to determine how the synthesis method and conditions affect the structure and adsorption capacity of the resulting samples. Three different synthesis routes were used: Slow evaporation (A), solvothermal synthesis using a PEG-1500 modulator (B), and green synthesis in water (C). All MOFs were characterized by various analytical methods: X-ray powder diffraction, elemental analysis, thermogravimetric analysis, scanning electron microscopy and volumetric nitrogen adsorption/desorption. In addition, the obtained MOFs were used to remove aqueous MB, CR, and MV, as well as to obtain nanostructured carbon materials.

## 2. Materials and Methods

### 2.1. Starting Materials

Methanol (MeOH, 99.9%), ethanol (EtOH, 98%), ethyl acetate, methylene chloride and dimethylformamide (DMF) were supplied by Sigma-Aldrich (Moscow, Russia); copper(II) sulfate pentahydrate (CuSO_4_·5H_2_O, ≥99.0%), copper(II) acetate monohydrate (Cu(CH_3_COO)_2_·H_2_O, ≥99.0%), 1,3,5-benzenetricarboxylic acid (H_3_BTC, 95.0%), triethylamine and a PEG-1500 modulator were purchased from Sigma-Aldrich (Moscow, Russia) and used without further purification. The purity of the solvents was determined by selective gas chromatography.

### 2.2. Adsorbates

Dyes methylene blue (МВ), Congo red (CR) and methyl violet (MV) having the molecular formulas C_16_H_18_N_3_SCl, C_32_H_22_N_6_Na_2_O_6_S_2_, and C_24_H_28_N_3_Cl, respectively, were chosen as adsorbates. They were purchased from Sigma-Aldrich with a solubility in water of 50, 10 and 50 g L^−1^ (20 °C) and a molecular weight of 319.85, 696.66, and 407.99 g mol^−1^, respectively. These dyes were chosen in this study because of their known strong adsorption onto solids. A stock dye solution was prepared by dissolving a precisely weighed dye in distilled water to a concentration of 200 mg L^−1^. Experimental solutions were obtained by diluting the stock dye solution in exact proportions to the required initial concentrations.

### 2.3. Synthesis of MOF

#### 2.3.1. Synthesis Using Slow Evaporation (A)

The following synthesis procedure is an optimization of the literary recipe [66]. 1,3,5-Benzenetricarboxylic acid (1 g, 4.8 mmol) was dissolved in 30 mL of a mixture of DMF/EtOH/H_2_O (1:1:1 ratio by volume) and added dropwise to Cu(CH_3_CO_2_)_2_·H_2_O (1.72 g, 8.6 mmol), which was dissolved in 30 mL of the same solvent mixture. Then, triethylamine (1.2 mL, 8.6 mmol) was added to completely deprotonate the linker and the resulting mixture was stirred for 30 min and slowly evaporated at room temperature for several weeks to obtain a blue precipitate. The precipitate was separated by centrifugation at 4500 rpm and washed several times with DMF. Then, the obtained sample was soaked in ethanol for several days with periodic replacement of the solvent and dried in vacuum (10^−3^ Torr, 80 °C, 10 h).

#### 2.3.2. Solvothermal Synthesis Using a PEG-1500 Modulator (B)

Copper trimesinate was also prepared using a modified solvothermal method based on the described procedure [67]. The modulator (PEG-1500), dissolved in DMF at a concentration of 0.7 mol L^−1^ under rapid stirring (900 rpm), was added to the same mixture of reagents and solvents that was used for procedure A. The resulting mixture was stirred for 30 min and placed in a stainless-steel autoclave with a Teflon liner. The autoclave was heated for 20 h at 120 °C, then was naturally cooled to room temperature. The resulting blue crystals were collected and purified as described above.

#### 2.3.3. Synthesis in Water (C)

NaOH (1.2 g, 3 mmol) was dissolved in a bidistilled water (50 mL) and 1,3,5-benzenetricarboxylic acid (2.1 g, 1 mmol) was added with heating to 80 °C and at constant stirring until a clear solution was obtained. CuSO_4_·5H_2_O (3.74 g, 1.5 mmol) was dissolved in water (20 mL) and slowly poured into the first solution, without stopping mixing. The blue precipitate was left in the vessel until self-cooling, and then it was separated in a centrifuge at 8000 rpm for 10 min, washed in a centrifuge under the same conditions with a bidistilled water. The precipitate was transferred to a flask with a tight stopper, into which dry methanol (50 mL) was added, stirred for 2 h and kept at room temperature for 12 h. The last procedure was repeated twice, separating the precipitate by centrifugation. The crystals separated from methanol were poured into dehydrated ethyl acetate (50 mL), stirred for 2 h, incubated for 12 h, repeating the procedure twice and the precipitate was also separated by centrifugation. The crystals, aged in ethyl acetate, were poured into anhydrous, stirred for 2 h and left for 12 h. The procedure was repeated twice, each time separating the target product by centrifugation. The precipitate was dried in air at 120 °C for 5 h, and then in vacuum at a residual pressure of 10^−6^–10^−8^ Torr at 150 °C for 6 h. The obtained violet crystals were stored in vacuum or in an atmosphere of dry argon. The weight of the collected and dried crystals is 4.86 g (95.5% based on H_3_BTC).

### 2.4. Characterization

Elemental analysis was performed on a Vario Micro cube analyzer (Elementar GmbH, Langenselbold, Germany), and Сu was determined on an AAS-3 atomic absorption spectrometer (Zeiss, Jena, Germany). X-ray powder diffraction (XRD) analysis was performed using an ARL™ X’TRA powder diffractometer (Thermo Fisher Scientific, Waltham, MA, USA) with CuKα radiation (λ_Cu_ = 1.54184 Å) in the range of 2θ = 5–80° with a scanning rate of 5°/min and temperature of 25 °C. Thermogravimetric analysis (TGA), performed simultaneously with differential scanning calorimetry (DSC) was carried out on a STA 409CLuxx synchronous thermal analyzer coupled to a QMS 403CAeolos quadrupole mass spectrometer (NETZSCH, Selb, Germany) in air at a rate of 10°/min in the range of 20–500 °C (powders, m = 0.3–0.4 g). Fourier-transform infrared (FTIR) spectra were performed on a Perkin-Elmer Spectrum 100 FTIR spectrometer using KBr pellets and Softspectra data analysis software (Shelton, CT, USA). Scanning electron microscope (SEM) analysis was conducted on a ZEISS Crossbeam 340 with an accelerating voltage of 3 kV (Jena, Germany). The detection of secondary electrons was carried out using an Everhart-Thornley detector (SE2), increasing the samples from 1.92 to 50 thousand times. The structure of thermolysis products was studied using a Tecnai G2 Spirit BioTWIN FEI high-resolution transmission microscope (Eindhoven, the Netherlands). The samples for high-resolution transmission electron microscopy (HRTEM) were prepared as follows: A powder suspension in hexane was applied onto a carbon-coated copper grid and the solvent was dried in air.

### 2.5. Determination of Kinetic and Thermodynamic Parameters of Thermal Decomposition

The activation energy was calculated using the Freeman-Carroll equation [68],

(1) $$\ln\left(\frac{dw}{dt}\right)W_{r}=\ln Z-\frac{E_{a}}{RT}$$

where *w* is the weight loss of the substance over time t, *W_f_* is the weight loss at the end of the process, *W_r_* is calculated as *W_r_* = *W_f_* − *w*, *Z* is the frequency factor, *E_a_* is the activation energy.

The entropy of activation (∆*S*), enthalpy of activation (∆*H*) and free energy of activation (∆*G*) were calculated using the following equations:

(2) ΔG=ΔH−TΔS

(3) $$\Delta H=E_{a}-RT$$

(4) $$\Delta S=2.303\left(\frac{Z_{h}}{kT}\right)R.$$

### 2.6. Measurement of Sorption Equilibrium Properties

The nitrogen adsorption/desorption isotherms were obtained at 77 K (liquid N_2_) using the AUTOSORB-1 system (Quantachrome, Boynton Beach, FL, USA) by the static volumetric method; before analysis the samples were degassed by heating at 150 °C for 12 h in vacuum. The Brunauer-Emmett-Teller surface area (S_BET_) was obtained from the amount of N_2_ physically sorbed at various relative pressures (P/P_0_), based on the linear part of the 6-point adsorption data at P/P_0_ = 0.02–0.10. The total pore volume (V_total_) was calculated by the Horvath Kawazoe method at P/P_0_ = 0.99. The micropore volume (V_micro_) was obtained by the Barrett-Joyner-Halenda adsorption and the t-plot methods, respectively. For assess the adsorption capacity, ultrahigh purity gases (99.995%) were used. The equilibrium values of the adsorbed gas volume were calculated as a function of the relative pressure in the system taking into account the weight of the sample and the volumes of various parts of the instrument.

### 2.7. Dye Equilibrium Adsorption Experiments

For equilibrium studies, a batch method was used because of its simplicity. A solution of the corresponding dye with a volume of 200 mL was placed in a 300 mL temperature-controlled beaker, thermostatically controlled at 283, 293, and 308 K on a magnetic stirrer, adjusting the rotation speed so that mixing was effective, but the air was not drawn into the liquid phase. When the solution reached a pre-determined temperature, an adsorbent (copper trimesinate synthesized by a method C, 0.1 g) was introduced and a timer was started. Every 15 min, 10 mL of a sorbent suspension in a dye solution was pipetted and centrifuged quickly; the concentration of residual dye was determined in the filtrate using a UV-visible spectrophotometer (Varian, Cary50) at λ_max_ = 492 nm (CR) [69], 664 nm (MB) [70], 584 nm (MV) [71], respectively.

The amount of the dye uptake by MOF in each flask was calculated by the following equations,

(5) $$q_{t}=\frac{\left(C_{0}-C_{t}\right)V}{m}$$

(6) $$q_{e}=\frac{\left(C_{0}-C_{e}\right)V}{m}$$

where *q_t_* and *q_e_* are the quantities (mg g^−1^) of the dye adsorbed on the adsorbent at time *t* and equilibrium, respectively; *C*_0_, *C_t_*, and *C_e_* are the dye concentrations in solution (mg L^−1^) initially, at time *t* and at equilibrium, respectively; m (g) and *V* (L) represent the amount of adsorbent and the volume of the dye solution, respectively.

The percentage removal of the dye (%) was calculated by the following equation:

(7) $$\mathrm{Removal}(\%)=\frac{\left(C_{0}-C_{e}\right)}{C_{0}}\times 100\%.$$

### 2.8. Studying the Kinetics of Adsorption

An adsorption model that describes the adsorption of a solute on a solid surface can be expressed as [72],

(8) $$\frac{dq}{dt}=k_{1}\left(q_{e}-q_{t}\right)$$

where *k*_1_ (min^−1^) is the rate constant of the pseudo-first order model.

After a definite integration from *t* = 0 to *t* = *t* and from *q* = 0 to *q* = *q_e_*, Equation (8) takes the form:

(9) $$\ln\left(q_{e}-q_{t}\right)=\ln q_{e}-k_{1}t.$$

The constant *k*_1_ can be determined experimentally from the slope of the linear plots ln(*q_e_* − *q_t_*) vs. *t*.

### 2.9. pH Experiments

To study the effect of pH on dye adsorption, a solution (80 mg L^−1^) of copper trimesinate was added to solutions (10 mg L^−1^) of dye. The initial pH was adjusted to 2–12 using HCl and NaOH. After shaking the suspensions for 120 min for an equilibrium time at a temperature of 300 K, they were filtered through 0.2 μm membrane filters and analyzed for the concentration of residual dye.

### 2.10. Study of Adsorption Isotherms

Two well-known isotherm equations, Langmuir and Freundlich, were used to interpret the obtained adsorption data.

#### 2.10.1. Langmuir Isotherm

The Langmuir adsorption isotherm suggests that adsorption occurs in certain homogeneous sites within the adsorbent, and has found successful application in many sorption processes of monolayer adsorption [73]. The Langmuir isotherm can be written in the form [74],

(10) $$q_{1}=\frac{q_{m}K_{L}C_{e}}{1+K_{L}C_{e}}$$

where *K_L_* is the Langmuir constant (L mg^−1^) associated with the affinity of the binding sites and the free energy of sorption; *q_m_* is the adsorption capacity expressing the dye concentration when a monolayer is formed on the sorbent (mg g^−1^).

For the Langmuir equation, the favorable nature of adsorption can be expressed in terms of the dimensionless separation coefficient of the equilibrium parameter, which is determined as follows:

(11) $$R_{L}=\frac{1}{1+K_{L}C_{0}}.$$

The *R_L_* values indicate that the type of isotherm is irreversible (*R_L_* = 0), favorable (0 < *R_L_* < 1), linear (*R_L_* = 1) or unfavorable (*R_L_* > 1) [75].

#### 2.10.2. Freundlich Isotherm

The Freundlich isotherm is an empirical equation used to describe heterogeneous systems [73]. The Freundlich equation is as follows,

(12) qe=KFCe1n

where *K_F_* is a constant showing the adsorption capacity of the adsorbent (mg^1−1/*n*^ L^1/*n*^ g^−1^), and *n* is an empirical constant related to the magnitude of the adsorption driving force.

The value of 1/*n* quantitatively determines the favorable adsorption and the degree of heterogeneity of the surface of the MOF. According to Halsey [76]:

(13) KF=qmC01n.

To determine the maximum adsorption capacity (*q_m_*), it is necessary to work with a constant initial concentration *C*_0_ and a variable adsorbent weights.

### 2.11. Determination of Thermodynamic Parameters of Dye Adsorption

Assuming that the activity coefficients are equal to unity at low concentrations (the meaning of Henry’s law), thermodynamic parameters (∆*G*^0^, ∆*H*^0^ ∆*S*^0^) were calculated using the following relations [77],

(14) $$K_{D}=\frac{q_{e}}{C_{e}}$$

(15) $$\Delta G^{0}=-RT\ln K_{D}$$

(16) $$\ln K_{D}=\frac{\Delta S^{0}}{R}-\frac{\Delta H^{0}}{RT}$$

where *K_D_* is the adsorbate distribution coefficient.

The parameters ∆*H*^0^ and ∆*S*^0^ can be calculated from the slope and intersection of the plot ln *K_D_* vs. 1/*T*, respectively.

### 2.12. Thermolysis Technique

In a typical experiment, a portion of the substance (0.6–0.8 g) is placed in a quartz tube with a minimum height of 10 cm and a diameter of 1 cm. The assembled unit is evacuated, filled with argon, heated at a rate of 50/min until a temperature of 400 °C is reached and kept at this temperature for 1 h. After the specified time, the unit is evacuated again, continuing to maintain the indicated temperature. Vacuuming is carried out until the walls of the external vessel are freed from volatile decomposition products. The resulting substance is cooled in vacuum to room temperature; the target product is removed in the form of a porous column with a height of 20–25 mm and crushed.

## 3. Results and Discussion

### 3.1. Preparation and Characterization of MOFs

Copper trimesinate samples were obtained using improved synthesis by slow evaporation, solvothermal synthesis using a PEG-1500 modulator, and synthesis at room temperature under green conditions with water. Based on the results of elemental analysis (Table 1) and TGA performed simultaneously with DSC, we proposed the empirical formulas Cu_3_(BTC)_2_·2DMF·2H_2_O and Cu_3_(BTC)_2_·2H_2_O for the obtained MOFs. A specific reaction mechanism was described previously [78].

FTIR spectra of MOFs were obtained using KBr pellets in the range of 500–4000 cm^−1^. The peak at 709 cm^−1^ corresponds to the formation of a bond between the metal and the carboxyl groups. The band at 1067 cm^−1^ is associated with the C–O–Cu stretching of the MOF. The band at 1620 cm^−1^ refers to the H–O–H vibration, which indicates the presence of crystalline water in the MOF. The peak at 3450 cm^−1^ is due to hydroxyl groups. There is no strong carbonyl peak at ~1710 cm^−1^, since the two resulting oxygen atoms on the carboxyl group tend to be equivalent, which leads to a delocalized electron cloud and the appearance of characteristic carboxylate peaks at 1424 and 1555 cm^−1^. The characteristic peaks in the IR spectra are in good agreement with the previously presented FTIR spectrum for MOF-199 [79].

The thermal behaviour of synthesized Cu_3_(BTC)_2_·2H_2_O is shown in Figure S1. The temperature ranges on the TGA curve completely coincide with the same intervals on the DSC curve. The thermal behaviour of MOF is characterized by the absence of phase transitions and shows that the thermal decomposition of the sample occurs in three stages. When a substance is heated in a helium flow with a temperature gradient of 5°/min, a small endothermic peak is detected in the range of 90–95 °C with a maximum at 93 °C, which corresponds to an energy consumption of 36.4 J g^−1^ and is associated with a weight loss of 5.6%, corresponding to the removal of two water molecules. With further heating, the substance remains stable up to 227 °С, followed by a second endothermic peak in the range of 227–245 °С with a maximum of 234.4 °С and an energy consumption of 92.4 J g^−1^ associated with a loss of 41.51% of the weight of the substance. The third endothermic peak is recorded in the range from 332.5 to 367.8 °С with a peak effect at 340.2 °С and the energy value of 96.67 J g^−1^, which is associated with the loss of 18% of the weight of the substance.

The thermal behaviour of Cu_3_(BTC)_2_·2DMF·2H_2_O is shown in Figure S2. Thermal decomposition of the sample occurs in three stages. The initial continuous weight loss up to 130 °C (5.01%) is due to the removal of physiosorbed solvent molecules from the framework, which corresponds to a theoretical loss (4.68%) for two water molecules. The second stage is observed between 130 and 286 °C and may be associated with the loss of two coordinated DMF molecules (calculated, 18.96 wt.%; found, 21.89 wt. %). Such a high-temperature removal of coordinated molecules indicates strong coordination interactions between Cu and the DMF molecule. Significant weight loss (38.64 wt.%) occurs at 360 °C and can be caused by complete damage of the BTC linker. This high thermal stability of metal-carboxylate MOFs is in good agreement with the data described previously [80,81].

The value of activation energy using the Freeman-Carroll Equation (1) for each stage of thermal decomposition of copper trimesinate synthesized in accordance with methods A and C was estimated from the plots of ln(*dw*/*dt*/*W_r_*) vs. 1/T (Figures S3 and S4). The thermodynamic parameters (∆*S*, ∆*H* and ∆*G*) were calculated using Equations (2)–(4). All copper trimesinate molecules have negative entropy, which indicates that decomposition reactions proceed with a lower rate than usual. The negative value of entropy also indicates that the activated complex has a more ordered and more rigid structure than reagents or intermediates. The negative values of activation entropies are compensated by the values of activation enthalpies, which leads to almost identical values of the free activation energy (Table 2).

**Figure 1:**
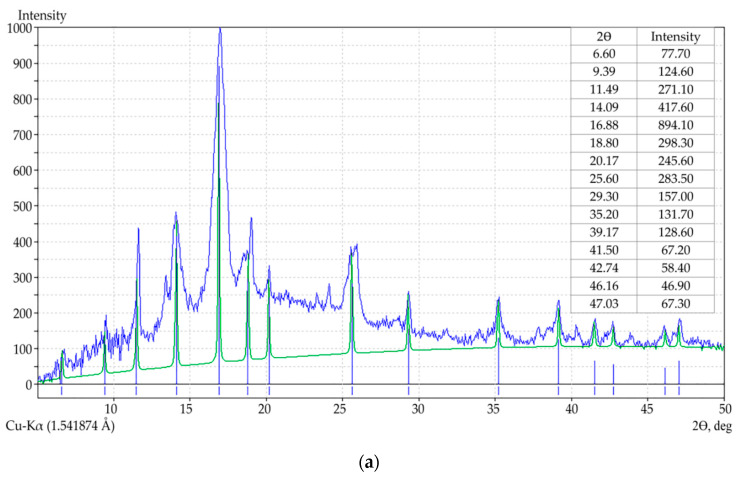

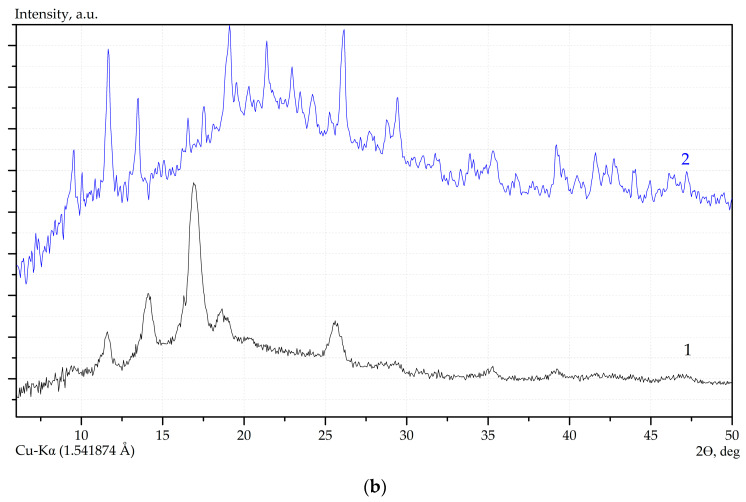
XRD pattern for copper trimesinate synthesized according to A (**a**), B (curve 1, (**b**)) and C (curve 2, (**b**)) methods.

The synthesized MOF structures were investigated using XRD. As can be seen from Figure 1, the main diffraction peaks have the 2θ values of 9.39, 11.49, 14.09, 16.88, 18.80, which are in good agreement with the literature data [82]. The resulting product is a mixture of crystalline (face-centered cubic) and X-ray amorphous phases. Partially, the presence of amorphous phases can be explained by the tendency of the ligand to stacking interaction and the formation of interpenetrated structures [83]. XRD measurements show polycrystalline copper trimesinate with a preferred orientation along the (111) direction [84]. In addition, the synthesis methods used in this study provide a reduced crystallization time and, therefore, the rapid generation of a large number of nuclei at an early stage of crystallization, therefore they do not always lead to a homogeneous crystalline sample.

**Figure 2:**
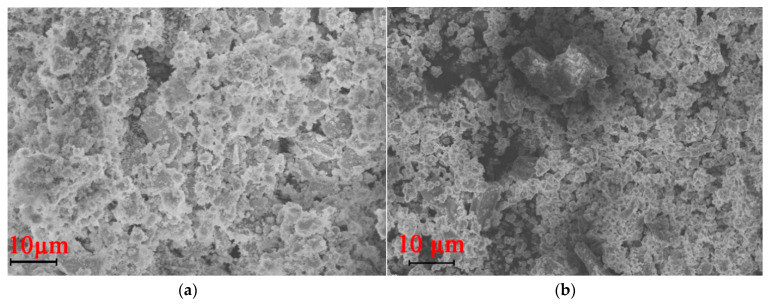

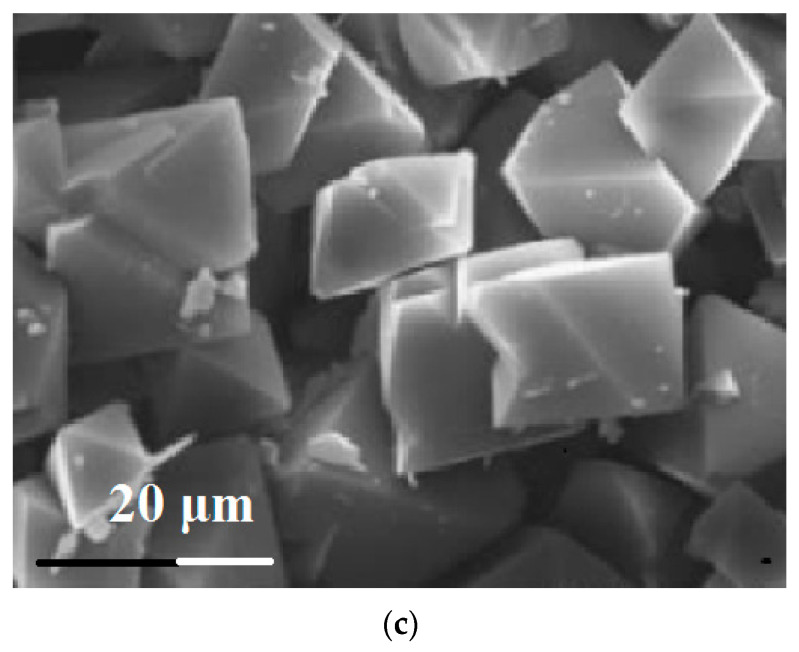
SEM images for MOFs synthesized according to the methods A (**a**), B (**b**), C (**c**).

SEM images showed the presence of highly dispersed MOF crystals and an unusual particle morphology, which was almost the same as previously reported [43,81]. It is important that the morphology of copper trimesinate depends on the method of its preparation. When using a mixture of solvents (DMF/EtOH/H_2_O) during the synthesis, it is quite difficult to get rid of the solvent, which ultimately affects the morphology of the product, in particular, smaller crystals are formed in the shape of pyramid (Figure 2a,b). The morphology of the products obtained under these conditions is similar to copper trimesinate synthesized by the aerosol method [82]. At the same time, when water is used as a solvent, pyramidal crystals of copper trimesinate are formed (Figure 2c), similar to crystals obtained by the solvothermal method [85], but they are less structured and are slightly worse formed during crystallization.

### 3.2. The Adsorption Capacity and Surface Area Analysis of Copper Trimesinate

**Figure 3:**
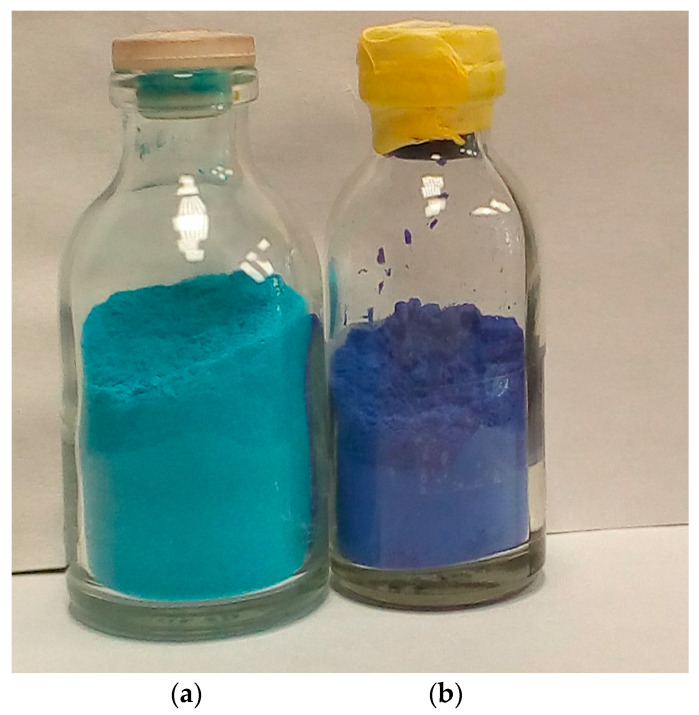
(**a**) Cu_3_(BTC)_2_·2H_2_O powder and (**b**) Cu_3_(BTC)_2_ obtained by heating to 180 °C for 30 min in vacuum.

The removal of two copper-bound water molecules provides access to open copper sites. This process and substitution of water with substrates or molecules of another solvent can be easily observed by the color change of the compound (Figure 3).

Before removing, the solvent molecules, in particular water, fill the axial coordination positions of the Cu^II^-paddlewheels. Once the coordinated water molecules are removed in vacuum, the material becomes sensitive to re-coordination of the ligand, so that irreversible decomposition can occur when exposed to air/moisture. This is usually true for all Cu-based MOFs, but not required for other metals. Therefore, only copper trimesinate with a low water content has a sorption capacity in aqueous solutions, since an absolutely dry preparation adsorbs water from a solution quickly and with a significant thermal effect and practically loses its sorption capacity.

The N_2_ adsorption-desorption isotherms of MOF at 77 K and pore size distribution curves are shown in Figure S5. The behavior of the nitrogen physiosorption isotherms confirms that the samples have permanent porosity and a microporous structure with a large surface area that corresponds to adsorption type I [67]. This behavior is typical of high-quality Cu_3_(BTC)_2_ material with little or no pore collapse or residual reactant [86]. The BET surface area and porosity properties of the samples are shown in Table 3. For copper trimesinate synthesized by method C, a high nitrogen adsorption of 590 cm^3^/g at 77 K was determined (sample with S_micropore_ = 1557.5 m^2^/g and S_external_ = 4.8 m^2^/g).

The large surface area of MOFs is an important feature that leads to high gas uptake capacity. Thus, the equilibrium values of the adsorbed volume of methane at 1 bar are 17.5 and 13.1 (296 К), 53.9 and 40.7 cm^3^/g (233 K) for samples synthesized according to methods A and B, respectively. The results showed a close correlation between the surface area of the sample and its CH_4_ uptake at 1 bar and 296 К. For hydrogen and carbon dioxide, the equilibrium values of the adsorbed volume of gases at 1 bar (294 K) are 4.06, and 66.5 cm^3^/g, respectively. In addition, in comparison with traditional porous materials such as activated carbon, the sample obtained by method C showed a higher adsorption capacity under similar adsorption conditions.

### 3.3. Dye Adsorption on MOF

**Figure 4:**
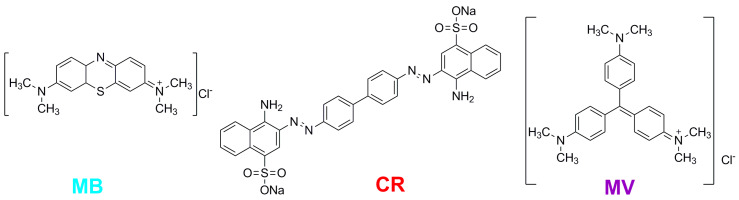
Dye chemical structures.

Methylene blue (MB), Congo red (CR), and methyl violet (MV) were used as model compounds (Figure 4).

The chemical and physical characteristics of the dye-exposed MOF powder confirm that the dye is present in the pores of the MOF without changing the crystal structure of the MOF. In particular, the powder XRD patterns of synthesized Cu_3_(BTC)_2_·2H_2_O and Cu_3_(BTC)_2_ with adsorbed dye showed that the crystal structure of MOF (face-centered cubic) was not affected by the dye adsorption process (Figure S6) [87].

**Figure 5:**
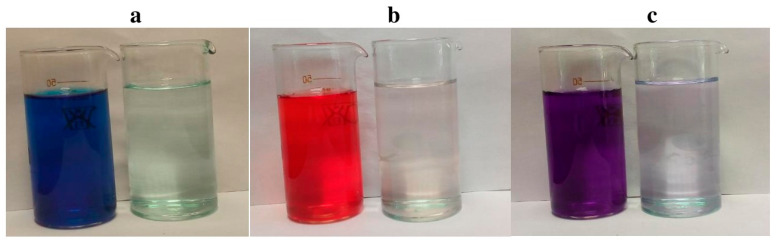
The color of the solution of dyes MC (**a**), CR (**b**) and MV (**c**) before and after adsorption.

The adsorption characteristics of MOF with respect to dyes were studied. The *q_e_* values of the MOFs for the dyes were completely different. In particular, these values were 396 and 394, 376 and 364, 366 and 362 mg g^−1^ at 283 and 308 K for CR, MB, and MV, respectively. The adsorption efficiency reaches almost 98%, 96.2% and 92% relative to CR, MB and MV, respectively, and leads to an almost complete discoloration of the dye solutions (Figure 5).

**Figure 6:**
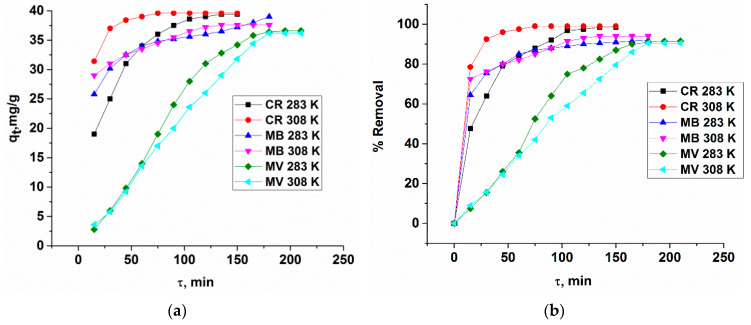
(**a**) Relationship between adsorbed quantities (*q_t_*) of the dyes and time. (**b**) Effect of contact time on the removal of the organic dyes by Cu_3_(BTC)_2_·H_2_O.

Figure 6a shows the quantities (*q_t_*) of the dye (CR, MB, MV) adsorbed on the adsorbent as a function of time t obtained using the Equations (5) and (6). As can be seen, the MOF adsorption abilities for dyes rapidly increased at the initial period of contact time, and then becomes slow with increasing contact time. In this case, rapid diffusion to the external surface is accompanied by rapid diffusion into the pores of the matrix, which leads to the rapid achievement of equilibrium. The adsorption capacities of MOF for dye removal were in the following order: CR > MB > MV, as shown in Figure 6b.

**Figure 7:**
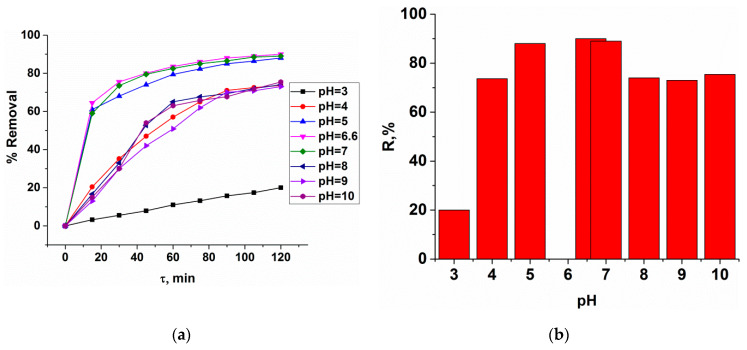
Dependence of the degree of MB adsorption on pH (**a**) and the maximum degree of MB adsorption on copper trimesinate depending on pH (t = 120 min) (**b**).

The maximum dye uptake on MOF occurred in the pH range 5–7 with a removal percent of ~90% (Figure 7), illustrating the excellent pH window offered by this adsorbent.

**Figure 8:**
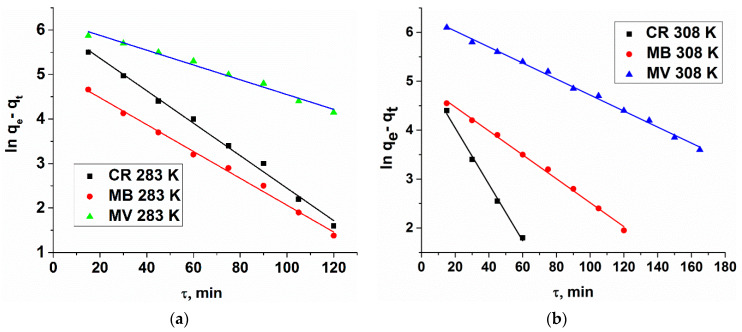
Modeling CR, MB, MV adsorption kinetics (linearized form) by Cu_3_(BTC)_2_·2H_2_O (pseudo-first order model) at T = 283 K (**a**) and 308 K (**b**).

The applicability of the pseudo-first-order kinetic model to the adsorption of the dyes by MOF was verified by fitting the experimental data to the model using least squares regression analysis, as shown in Figure 8. The pseudo-first-order adsorption rate constants calculated graphically are listed in Table 4.

The Langmuir and Freundlich isotherms of dyes by MOFs are shown in Figures S7 and S8. The adsorption isotherm is characterized by certain coefficients, the values of which express the surface properties and affinity of the adsorbent, and can also be used to determine the maximum adsorption capacity. Table 5 summarized the coefficients of isotherms at different temperatures. It can be seen that most of *R*^2^ values exceed 0.9 for all isotherm models. This suggests that all models are in close agreement with experimental results. Based on the coefficients of the Langmuir isotherm model, the R*_L_* values for dye adsorption on MOF at various temperatures were less than 1 and greater than zero, which indicates favorable adsorption. The *k_L_* values were 0.93, 2.16 and 3.12 L mg^−1^ at 290, 300 and 310 K, respectively, revealing that dye adsorption on MOF increases with temperature. The results obtained with the Freundlich isotherm show that the values of k_F_ and 1/n increased, and decreased with the increasing of temperatures, respectively. The 1/n values were between 0 and 1, indicating that dye adsorption on MOF was favorable under the conditions studied.

**Figure 9:**
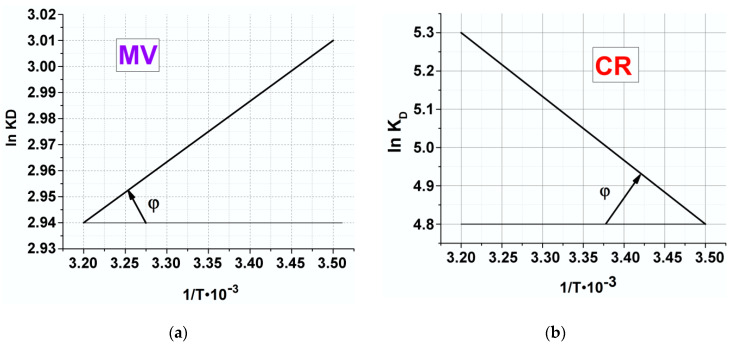

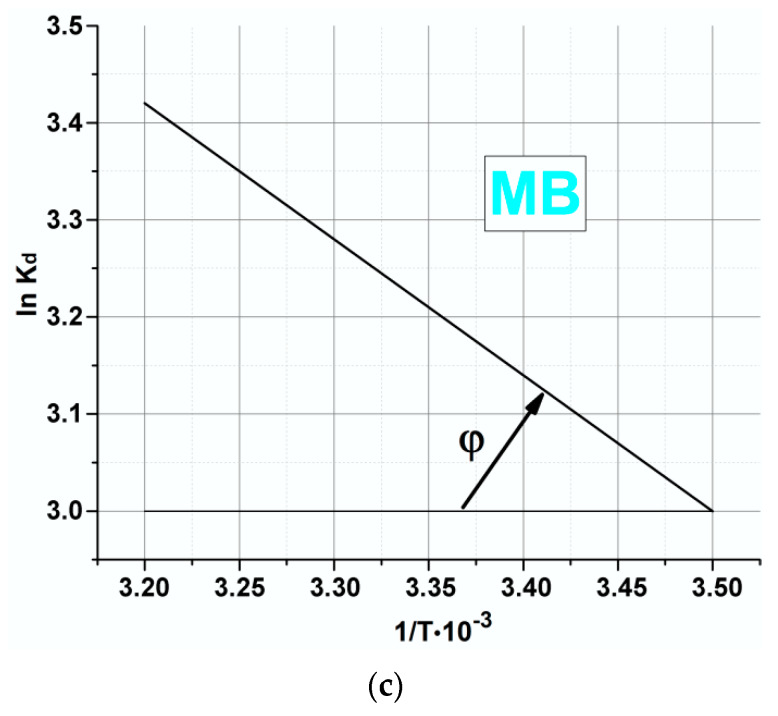
Plot of lnK versus 1/T for estimation of thermodynamic parameters for MV(**a**), CR (**b**), and MB (**c**) and adsorption onto Cu_3_(BTC)_2·_2H_2_O.

For estimation of thermodynamic parameters for CR, MB and MV adsorption onto Cu_3_(BTC)_2_·2H_2_O, the plots of lnK_D_ versus 1/T were used (Figure 9).

As it can be seen from Table 6, ∆*G*^0^ values at the temperatures of 290, 300 and 310 K are negative. These indicate that the adsorption process was a spontaneous process. The decrease in ∆*G*^0^ with the increase of temperature indicates more efficient adsorption at higher temperature. The positive ∆*H*^0^ value confirms that the adsorption process is endothermic for MV, which is an indication of the existence of a strong interaction between MOF and MV. For MV travel through solution and reach the adsorption sites, it is necessary for them first to be stripped out (at least partially) of their hydration shell, this process requires energy input. Thus, the positive value of ∆*H*^0^ indicates that the adsorption increased with temperature. Moreover, the positive value of ∆*S*^0^ indicates that the degrees of freedom increased at the solid-liquid interface during the adsorption of dyes onto MOF and reflected the affinity of MOF toward dyes ions in aqueous solutions and may suggest some structural changes in adsorbents [88].

### 3.4. Thermolysis of MOFs

**Figure 10:**
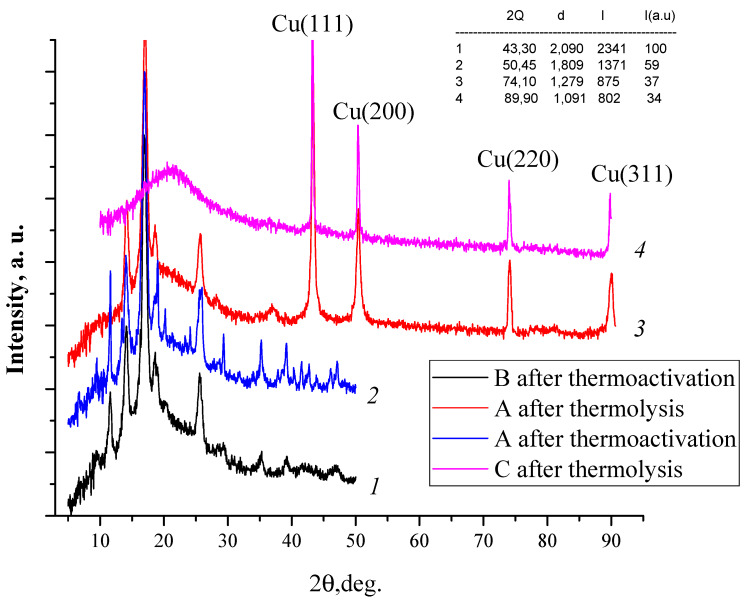
XRD patterns of copper trimesinates B (*1*), A (*2*) and the Cu@C (*3, 4*) samples obtained by thermolysis of A (*3*) and C (*4*) compounds at the different conditions (*3*—400 °C, 2 h, in vacuum, heating rate 2 °C/min; *4*—400 °C, 2 h, in self-generated atmosphere, heating rate 7 °C/min).

Thermolysis of the obtained MOFs was carried out at 400 °C to obtain carbon materials. Figure 10 depicts the XRD pattern of the Cu@C samples. The main XRD peaks of Cu@C at 2θ = 43.25, 50.35, 74.05, and 89.80 deg. correspond to the (111), (200), (220), and (311) planes, respectively. The peak intensity indicates a high degree of crystallinity and the absence of copper oxide in the structure of the product (high purity of the material). Interestingly, distinct synthetic conditions of thermolysis including temperature, medium and precursor source render morphology-controlled Cu@C materials.

**Figure 11:**
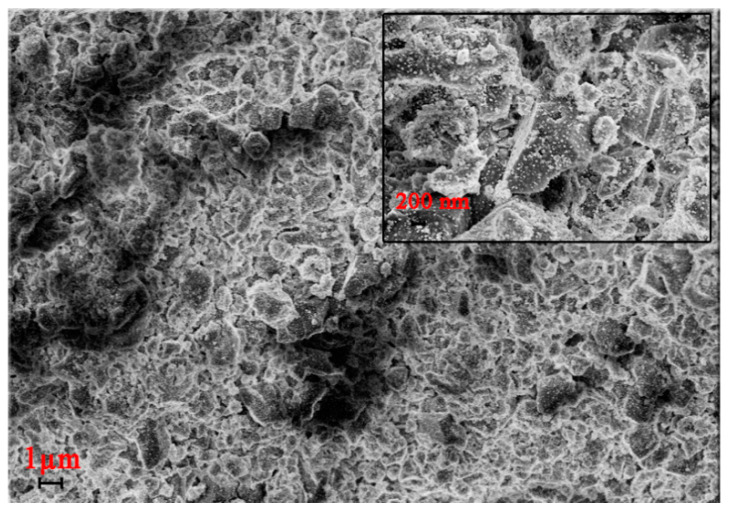
SEM image of thermolysis products of copper trimesinate obtained by method A.

In accordance with Scherer formula and SEM micrograph, during thermolysis of copper trimesinate, copper nanoparticles of 18–20 nm in size are formed. The thermolysis products at 400 °C have the form of dense blocks of various shapes of a homogeneous structure (Figure 11). They have a different shape, because during the preparation of the sample, it is treated with ultrasound, which breaks sample into separate pieces.

**Figure 12:**
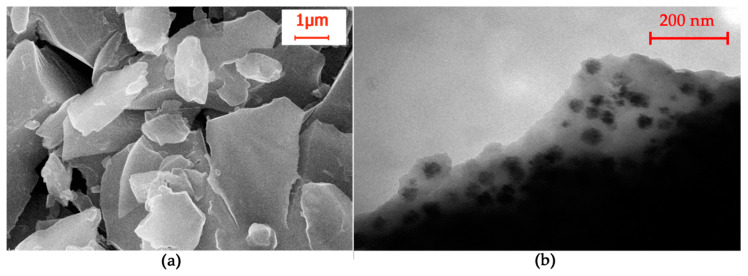
SEM; (**a**) and HRTEM (**b**) of the thermolysis product of copper trimesinate synthesized according method C.

The thermolysis products of copper trimesinate obtained by method C have a dense structure of black color; the morphology in the SEM image (Figure 12a) can be described as blocks of various shapes with a shell fracture. A TEM image (Figure 12b) allows one to determine electron-opaque metal particles ranging in size from 18 to 46 nm, the average size calculated according to the Scherer formula is 32.4 nm, enclosed in a carbon shell. The range of sizes of copper particles in the XRD pattern and in the TEM image satisfactorily coincides.

## 4. Conclusions

Therefore, three different synthesis pathways: Slow evaporation (A), solvothermal synthesis using a PEG-1500 modulator (B), and green synthesis in water (C) were successfully used for the efficient and reliable synthesis of thermally stable microporous copper trimesinate MOFs. It is shown that the synthesis conditions affect the structure and properties of MOF, the structures of which were confirmed by FTIR, XRD, SEM, and TGA methods. Samples have a permanent porosity and microporous structure with a large surface area corresponding to the adsorption type I. MOF showed good ability to adsorb organic dyes, such as methylene blue (МВ), Congo red (CR) and methyl violet (MV). Dye adsorption followed pseudo-first-order kinetics and was described by the Langmuir and Freundlich isotherms, for which the isotherm constants were determined. Thermodynamic parameters, such as changes in the free energy of adsorption (∆*G*^0^), enthalpy (∆*H*^0^), and entropy (∆*S*^0^), were calculated. High purity Cu@C carbon materials were obtained by thermolysis of copper trimesinate.

## Figures and Tables

**Table 1:** The results of an elemental analysis for samples synthesized under different reaction conditions.

Sample (Estimated Formula)	Method of Synthesis	Elemental Content, wt. % (Found/Calc.)		
		C	H	Сu
Cu_3_(BTC)_2_·2DMF·2H_2_O	A	36.92/36.81	3.63/3.07	24.14/24.35
Cu_3_(BTC)_2_·2DMF·2H_2_O	B	34.63/36.81	3.54/3.07	24.95/24.35
Cu_3_(BTC)_2_·2H_2_O	C	35.74/33.72	1.21/1.56	31.54/29.74

**Table 2:** Kinetic and thermodynamic parameters of the decomposition reaction of copper trimesinate synthesized according to methods A and C.

	Z, s^−1^		E_a_, kJ/mol		∆*H*, kJ/mol		∆*G*, kJ/mol		∆*S*, J/mol	
	A	C	A	C	A	C	A	C	A	C
Stage 1	0.25	0.17	85.68	71.09	82.7	88.11	331.7	−182.9	−698.04	701.1
Stage 2	0.18	0.9	132.9	140.7	128.64	137	−232	−106.6	−703.03	−690
Stage 3	0.03	0.02	145.15	96.26	140.06	91.17	581.42	534.37	−720	−723

**Table 3:** Specific surface, area volume and pore distribution of copper trimesinate.

Sample	S_BET_ (m^2^/g)	V_total_ (cm^3^/g)	V_micro_ (cm^3^/g)	Average Pore Radius (Å)
A	1348	0.7	0.51	14
B	830	0.9	0.31	28
C	1562.3	0.85	0.61	10.9

**Table 4:** Pseudo-first-order adsorption rate constants calculated graphically.

Dye	283 K	308 K
CR	0.47	0.9
MB	0.36	0.41
MV	0.18	0.27

**Table 5:** Parameters of the Langmuir and Freundlich isotherms for dye adsorption on Cu_3_(BTC)_2_·2H_2_O.

Model	CR	MB	MV
Langmuir	*q_m_* = 170.9 mg g^−1^	*q_m_* = 173.8 mg g^−1^	*q_m_* = 34.9 mg g^−1^
	*K_L_* = 1 L mg^−1^	*K_L_* = 1.0034 L mg^−1^	*K_L_* = 0.7 L mg^−1^
	*R*^2^ = 0.98	*R*^2^ = 0.97	*R*^2^ = 0.95
Freundlich	*q_e_* = 30 mg g^−1^	*q_e_* = 80 mg g^−1^	*q_e_* = 125 mg g^−1^
	*K_F_* = 0.3	*K_F_* = 0.1	*K_F_* = 0.57
	*R*^2^ = 0.98	*R*^2^ = 0.97	*R*^2^ = 0.95

**Table 6:** Thermodynamic parameters of the adsorption process.

Dye	∆*G*^0^ (kJ mol^−1^)		∆*H*^0^_293_ (kJ mol^−1^)	∆*S*^0^_293_ (J mol^−1^)
CR	283 K	−11.288	−10.31	11.5
	293 K	−12.3		
	308 K	−11.72		
MB	283 K	−7.058	−6.6	16
	293 K	−7.8		
	308 K	−8.8		
MV	283 K	−7.082	2.9	4.7
	293 K	−7.31		
	308 K	−7.5		

## Supplementary Materials

The following are available online at https://www.mdpi.com/2073-4360/12/5/1024/s1, Figure S1: TGA and DSC curves for Cu_3_(BTC)_2_·2H_2_O synthesized by method C, Figure S2: DSC and TGA curves of Cu_3_(BTC)_2_·2DMF·2H_2_O synthesized by method A, Figure S3: Plot of Freeman-Corrols equation ln(dw/dt)/dw_r_ v/s 1/T for copper trimesinate synthesized by method A: stages 1 (a), 2 (b), 3 (c), 4 (d), Figure S4: Plot of Freeman-Corrols equation ln(dw/dt)/dw_r_ v/s 1/T for copper trimesinate synthesized by method C: stages 1 (a), 2 (b), 3 (c), 4 (d), Figure S5: N_2_ adsorption-desorption isotherms and the pore size distribution curves for copper trimesinate synthesized by methods A (a,b), B (c,d) and C (e,f), Figure S6: XRD patterns for copper trimesinate with absorbed MV at 35 °C (*1*), copper trimesinate with absorbed MV at 10 °C (*2*), copper trimesinate (*3*), and MOF-199 calculated, Figure S7: Langmuir linear isotherms for CR, МВ and MV adsorption onto Cu_3_(BTC)_2_·2H_2_O at Т = 283 К, Figure S8: Freundlich isotherm for CR, MB and NV adsorption onto Cu_3_(BTC)_2_·2H_2_O at T = 283 K.
