# Supplementary material for: Synthesis of Copper(II) Trimesinate Coordination Polymer and Its Use as a Sorbent for Organic Dyes and a Precursor for Nanostructured Material
Source: Polymers (Basel). 2020 May 1;12(5):1024. doi: 10.3390/polym12051024 (PMC7284901; doi:10.3390/polym12051024)
Supplement: Supplementary file 1 [file polymers-12-01024-s001.pdf]

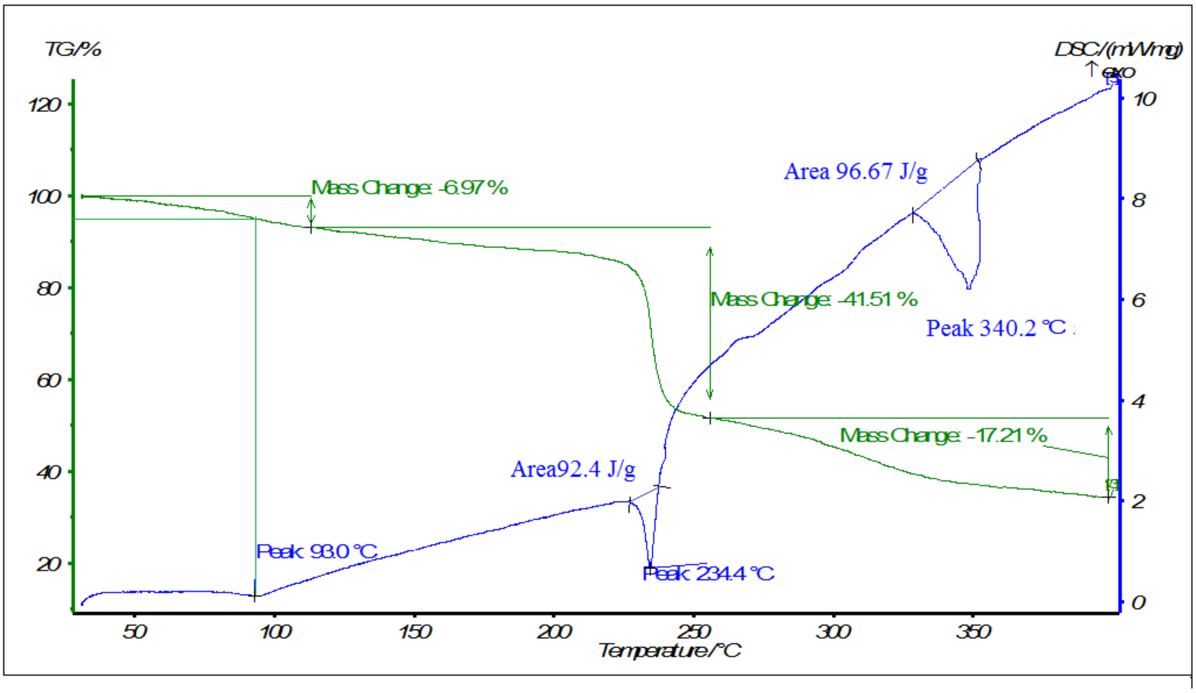


**Figure 1S.** TGA and DSC curves for $Cu_3(BTC)_2{\cdot}2H_2O$ synthesized by method C.

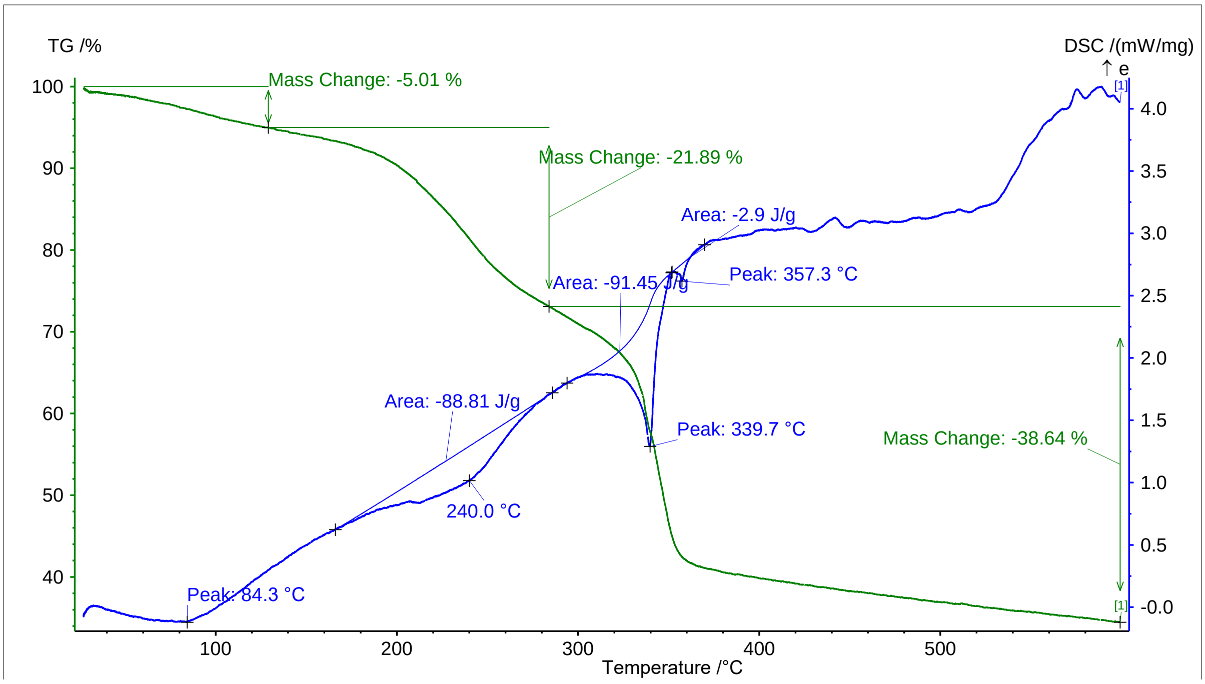


**Figure 2S.** DSC and TGA curves of $Cu_3(BTC)_2{\cdot}2DMF{\cdot}2H_2O$ synthesized by method A.

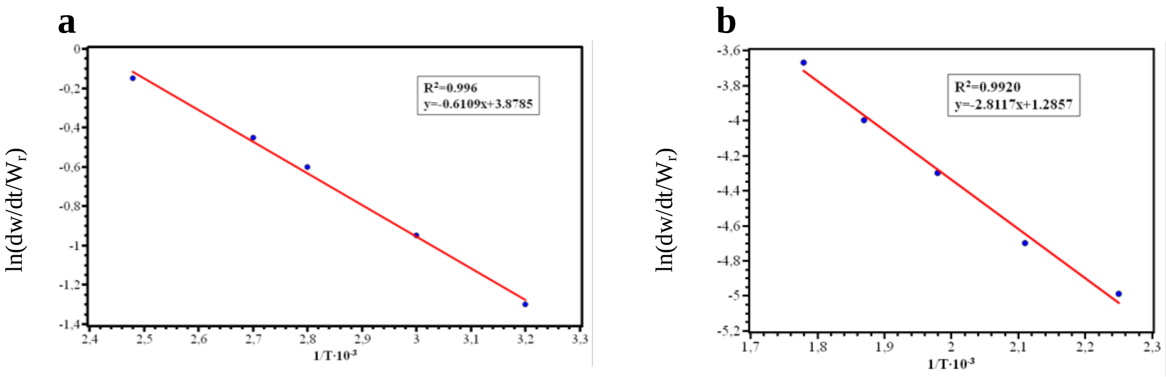

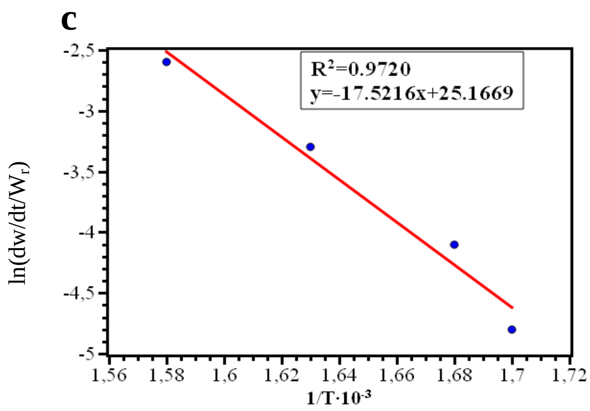


**Figure 3S**. Plot of Freeman-Corrols equation ln(dw/dt)/$dw_r$ v/s 1/T for copper trimesinate synthesized by method A: stages 1 (**a**), 2 (**b**), 3 (**c**).

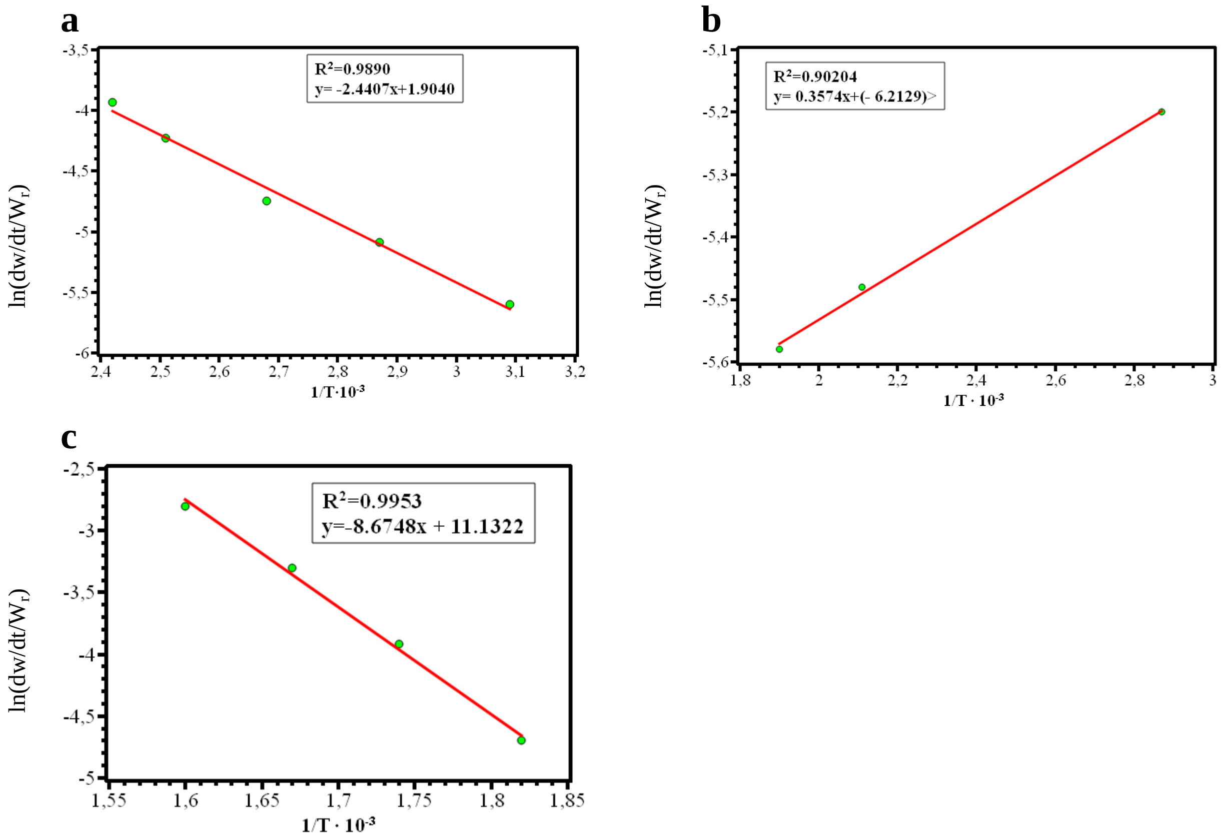


**Figure 4S**. Plot of Freeman-Corrols equation ln(dw/dt)/$dw_r$ v/s 1/T for copper trimesinate synthesized by method C: stage 1 (**a**), 2 (**b**), 3 (**c**).

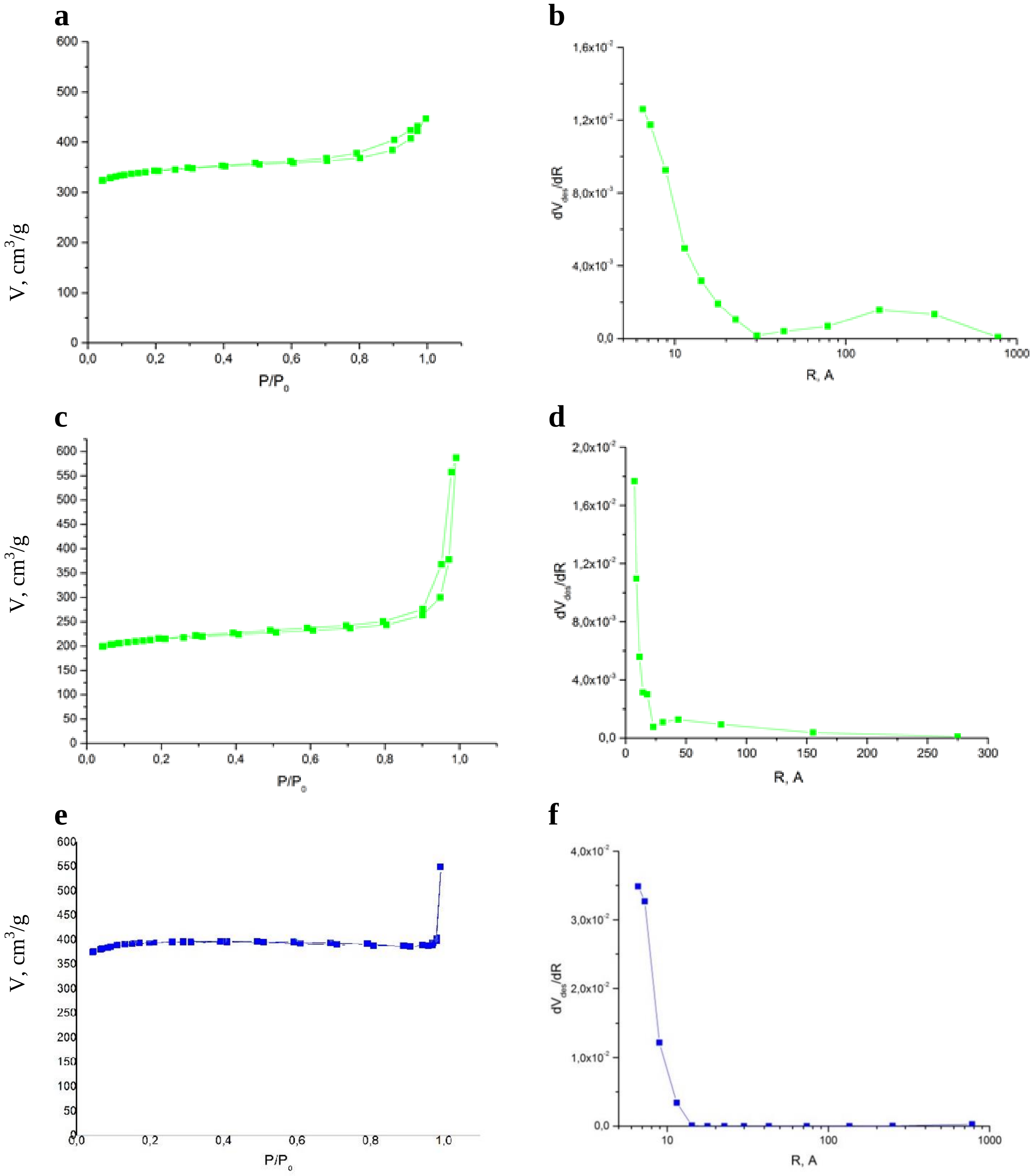


**Figure 5S.** $N_2$ adsorption-desorption isotherms and the pore size distribution curves for copper trimesinate synthesized by methods A (**a**, **b**), B (**c**, **d**) and C (**e**, **f**).

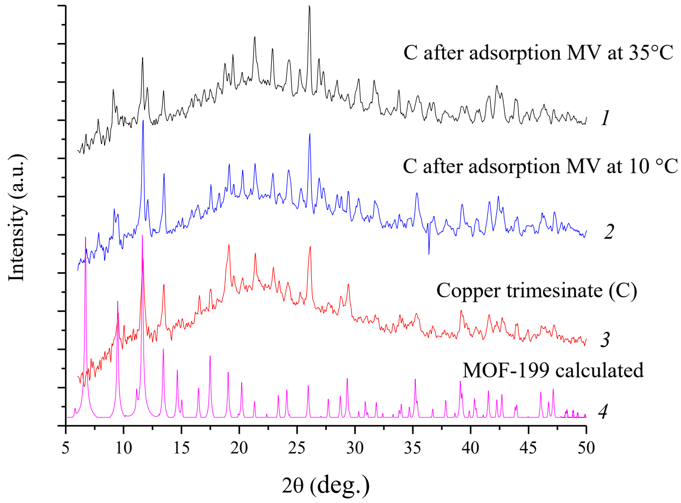


**Figure 6S.** XRD patterns for copper trimesinate with absorbed MV at 35 $^0$C (*1*), copper trimesinate with absorbed MV at 10 $^0$C (*2*), copper trimesinate (*3*), and MOF-199 calculated.

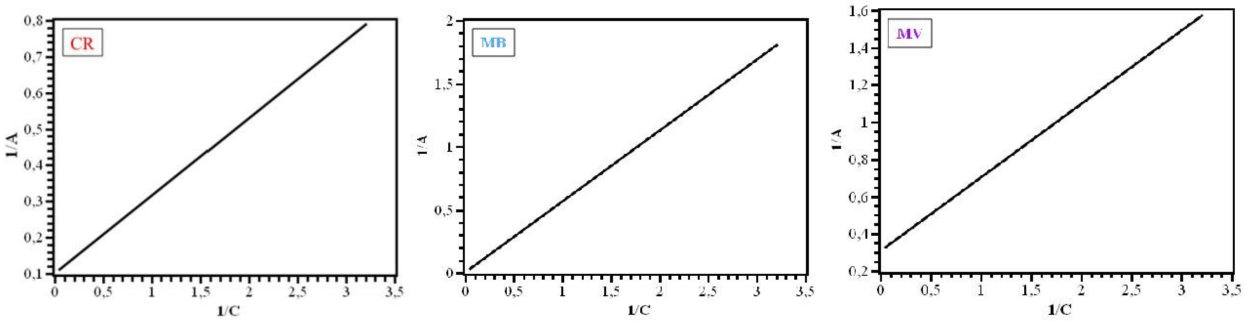


**Figure 7S.** Langmuir linear isotherms for CR, MB and MV adsorption onto $Cu_3(BTC)_2 \cdot 2H_2O$ at T = 283K.

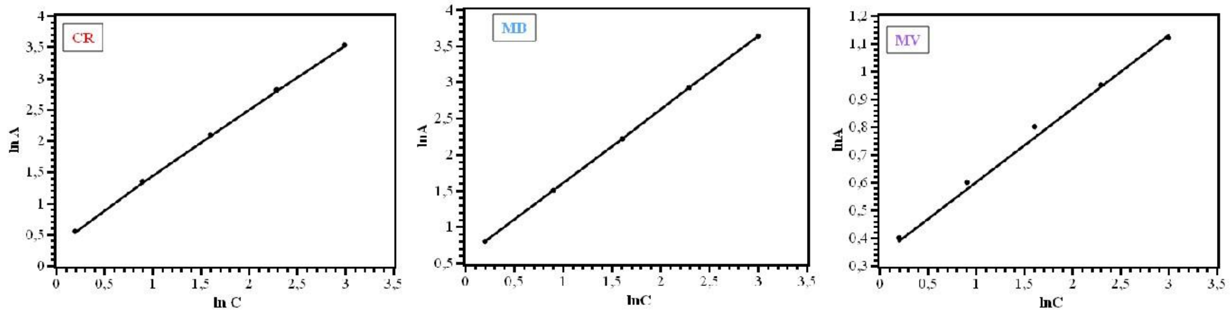


**Figure 8S**. Freundlich isotherm for CR, MB and MV adsorption onto $Cu_3(BTC)_2 \cdot 2H_2O$ at T = 283K.
